# Molecular characterisation of plasma membrane-derived vesicles

**DOI:** 10.1186/s12929-015-0174-7

**Published:** 2015-08-11

**Authors:** Samuel S. Antwi-Baffour

**Affiliations:** Department of Medical Laboratory Sciences, School of Biomedical and Allied Health Sciences, College of Health Sciences, University of Ghana, P. O. Box KB 143,, Korle-Bu, Accra, Ghana

**Keywords:** PMVs, Flow-cytometry, Characterisation, Proteins, Membrane, Microparticles

## Abstract

Plasma membrane-derived vesicles (PMVs) are released into circulation in response to normal and stress/pathogenic conditions. They are of tremendous significance for the prediction, diagnosis, and observation of the therapeutic success of many diseases. Knowledge of their molecular characteristics and therefore functional properties would contribute to a better understanding of the pathological mechanisms leading to various diseases in which their levels are raised. The review aims at outlining and discussing the molecular characteristics of PMVs in order to bring to the fore some aspects/characteristics of PMVs that will assist the scientific community to properly understand the role of PMVs in various physiological and pathological processes. The review covers PMVs characterisation and discusses how distinct they are from exosomes and endosomes. Also, methods of PMVs analysis, importance of proper PMV level estimation/characterisation, PMVs and their constituents as well as their therapeutic significance are discussed. The review concludes by drawing attention to the importance of further study into the functions of the characteristics discussed which will lead to understanding the general role of PMVs both in health and in disease states.

## Introduction

Plasma membrane-derived vesicles (PMVs), also referred to as microparticles (MPs), microvesicles (MVs) or more rarely ectosomes, are sub-membrane fragments shed from the plasma membrane of cells during cell growth, activation, proliferation, senescence, apoptosis and when stimulated [1]. Many cell types including leukocytes, platelets and endothelial cells release these small membrane fragments. PMVs therefore originate from cells which are surrounded by plasma membranes [2].

The presence of basal levels of PMVs is common in healthy individuals and an increase in their release although a controlled event, is a hallmark of cellular alteration [1]. Therefore, pharmacological modulation of circulating PMV concentrations could become a major therapeutic target in the future [3]. Various publications have discussed PMVs in relation to various disease in which their levels are raised [4]. This suggest the importance of PMVs as a key role player in various cell processes rather than just inert bi-products of cellular activation and therefore in disease pathogenesis [5].

### PMV characterisation

PMVs shed from both normal and cancerous cells may serve as a means of intercellular communication as they carry proteins, lipids and nucleic acids derived from the host cell [6, 7]. Their isolation and analysis from blood samples has the potential to provide information about the state and progression of malignancy in terms of cancer [6, 7]. PMVs are also likely to prove of great clinical importance as biomarkers for a variety of disease states in other words of potential diagnostic value as well as a therapeutic tool [8]. However, a standardized protocol for isolation has not yet been agreed upon [9]. It is often unclear what the content of the isolates are and whether the isolated PMVs, were present *in vivo* or whether they were created during the isolation procedure [10]. Vesicular structures, which are sized from 1 μm down to 50 nm, are present in isolates of many body fluids. Isolates of PMVs sometimes contain different populations that differ in size and shape, which indicates that methods of isolation and determination of the number of PMVs in the peripheral blood need to be elaborated and improved upon [9, 10].

A difficulty in the study of PMVs has been comparing results between laboratories. Since PMVs exist as heterogeneous species and express several different cell surface markers, which can vary according to the cell of origin and depending on the stimulus that caused their release, it is unlikely that any single marker will efficiently label all PMVs [10]. Thus, the need for precise and reproducible quantification is necessary if PMVs are to be a reliable marker for diagnosis. To date, the measurement and detection of levels of PMVs in various conditions have not translated into therapeutic or diagnostic strategies in the management of disease conditions in which they have been shown to be relevant [9, 10]. However they have helped to shift the understanding of the pathophysiological mechanisms of several diseases such as sickle cell and thrombotic thrombocytopenic purpura [8]. The detection of chronically elevated levels of circulating PMVs in patients with sickle cell disease provides an insight into the chronic endothelial attack that characterises this condition, and may provide an important tool in measuring the protective effects of therapeutic interventions in an early and non-invasive manner [11].

### Exosomes, endosomes and PMVs are distinct

**Fig. 1:**
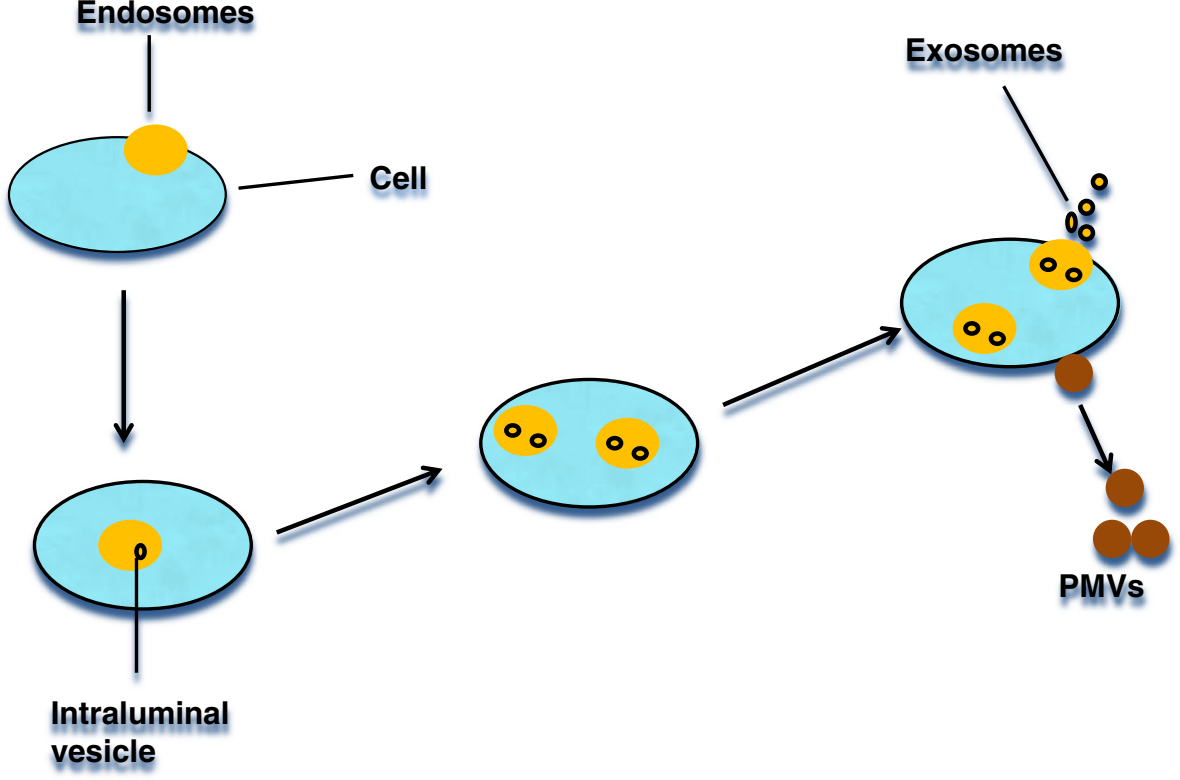
Schematic presentation of modes of emission of PMVs vs. Exosomes. This figure shows the formation of an endosome by invagination. By inward blebbing of the endosomal membrane, intraluminal vesicles are formed. Endosomes containing intraluminal vesicles are called multivesicular bodies (MVBs). Cells release the contents of their MVB when the membrane of the MVB fuses with the plasma membrane and in contrast to exosomes, PMVs are formed by major structural rearrangements of the cytoskeleton and are ‘budded’ off from the outer cell membrane

Exosomes were described in the early 1980s as 5’-nucleotidase activity-containing vesicles and to range in size from 30 to 90 nm (Fig. 1). In contrast to PMVs, exosomes are preformed membrane vesicles, which are stored in cellular compartments named multivesicular bodies (MVB), and secreted when the MVBs fuse with the cell membrane [12]. Many eukaryotic cells release vesicles like exosomes spontaneously or under appropriate stimulation. They were shown to be involved in the removal of the transferrin receptor from maturing reticulocytes by ‘invagination’ blebbing. These blebs (called endosomes) pinch off very small (30–90 nm) ‘intraluminal’ vesicles [12, 13]. These endosomes containing ‘intraluminal vesicles’ are stored in the MVB and once the ‘intraluminal’ vesicles become secreted, i.e. after membrane fusion of MVB and the surrounding plasma membrane - they are called exosomes [13].

Endosomes are therefore membrane-bound vesicles, formed via a complex family of processes collectively known as endocytosis, and found in the cytoplasm of virtually every animal cell. The basic mechanism of endocytosis is the reverse of what occurs during exocytosis or cellular secretion. It involves the invagination of a cell’s plasma membrane to surround macromolecules or other matter diffusing through the extracellular fluid [14]. The encircled foreign material is then brought into the cell, and following a pinching-off of the membrane (termed budding), is released into the cytoplasm in a sac-like vesicle [15].

PMVs and exosomes/endosomes are morphologically distinct vesicle populations [16]. A recent study revealed that protease-containing PMVs shed from tumor-cell lines appear to be rather heterogeneous in size and shape as opposed to exosomes, which are a more uniform population of vesicles [17]. The same study also showed that microvesicles sediment at lower speeds relative to exosomes, which pellet at 100,000 g. Also, exosomes from red blood cells contain the transferrin receptor found in reticulocytes, which is absent in mature erythrocytes and are therefore not seen in PMVs. Again, dendritic cell-derived exosomes express MHC I, MHC II, and costimulatory molecules and have been proven to be able to induce and enhance antigen-specific T cell responses *in vivo*. Exosomes can also be released into urine by the kidneys, and their detection might serve as a diagnostic tool. In fact, urinary exosomes may be useful as treatment response markers in prostate cancer. Furthermore, exosomes are remarkably more stable in bodily fluids than PMVs, strengthening their case as good reservoirs for disease biomarkers.

### Methods of PMV analysis

Although several methods for analysis of PMVs have been reported, conventional Flow-cytometry and ELISA are the most widely adopted [18]. Florescence Activated Cell sorting (FACS) (flow cytometry method) is the commonly used method for analysing the number, size, and properties of PMVs [17, 18]. With flow cytometry, it is possible to determine the number of events (forward scattering) and the density of the events (side scattering) allowing quantification of the PMVs identified in each case [19]. However, because of the sizes, detection is limited by ‘noise’ in the flow cytometer as conventional FACS instruments are mostly designed to measure cells, which are 10-to 100-fold greater in diameter than PMVs [17, 18]. Antibodies to cell surface molecules allow the identification of specific PMV subpopulations as in the case of FACS analysis of cells, but because of the size of PMVs, the amount of any surface marker is drastically reduced in comparison to intact cells, thereby limiting detection [20]. In addition to proteins, the presence of phosphatidylserine on the outer leaflet of the PMV plasma membrane allows binding of annexin V, which is also used to identify and enumerate the PMVs via FACS [17, 18].

Some investigators identify PMVs with a forward angle light scatter smaller than the 1-1.5 μm latex beads most instruments use as an internal standard whilst others also employ a lower size limit of 100 nm and any particles detected by the flow cytometer under this size are not considered true PMVs leading to underestimation [21]. Using the upper size (1.5um) limit criteria could also lead to an underestimation of true PMV numbers as some investigators consider PMVs to be up to 2 μm in size [17, 18]. As different flow cytometers are used to analyse PMVs, it may be possible that the flow cytometer technique and the way they are used in terms of interpreting results could have a greater part to play in PMV analysis. For example, laser alignments can differ and different instruments capture PMVs in different ways. Bench top flow cytometers such as FACSCalibur have a fixed laser in constant alignment needing no human adjustment whereas FACSVantage SE uses removable lasers, which need manual adjustment [19].

For capture, FACSCalibur systems use a device referred to as a catcher tube to sort PMVs. The catcher moves in and out of the sample stream to collect a population of desired PMVs, with a maximum rate of 300 per second [17–19]. The laser alignment and stream velocity are fixed, making the time it takes for PMVs to travel from the laser to the catcher tube constant. Conversely, the FACSVantage SE isolates a cell of interest by vibrating the stream. The sample stream vibrates and separates the sample into drops, with the distance between drops being fixed. When the sheath velocity and the vibration speed are constant, the pattern of drop formation is fixed, allowing the distance between drops to be calculated followed by PMV isolation [19]. Even though no known studies appear to have used FACSVantage SE to analyse PMVs, highlighting such differences alerts investigators prior to undertaking experiments that flow technology and its incorrect implementation could lead to erroneous results.

An alternative method for identifying PMVs involves binding assays in a solid-phase or microtitre plate format [20]. In this approach, antibodies to cell surface molecules can capture particles for subsequent detection by another antibody or a functional assay [22]. While such assays can assess the total amount of PMV-related material in a specimen, they cannot provide information on the number or size of particles [20, 22].

PMVs analysis can be impacted not only by type of assay, but also by the manner in which sample (blood) is collected and processed, including sampling site, needle diameter, centrifugation, re-suspension and washing of the isolated PMV pellet [18, 19]. Also it is important to ensure that platelets are removed, since they interfere with PMV detection, giving false positive signals [23]. At present, PMV analysis is constrained by lack of standardized PMV assays [24]

### Importance of proper PMV level estimation/characterisation

PMVs constitute a dynamic circulating storage pool by themselves, able to induce vascular responses to pro-apoptotic, inflammatory, or thrombotic stimuli [24]. Therefore, the clinical background or outcome associated with elevated PMV levels or accelerated clearance should be taken into close consideration in deciphering their multiple effects [25]. For instance, misleading quantification or phenotypes could result from accelerated degradation by secretory phospholipase A2, interactions with the vascular wall, or trapping in cell–cell aggregates or within the thrombus [26].

An additional complexity recently observed was that circulating PMVs may bear antigens from different cellular origin, pointing to multiple transcellular PMVs-mediated exchanges [27]. Antigens specifically expressed during cell activation could prove useful in identifying the various pathways of PMV release and discriminating underlying pathologies and associated damages [8]. The circulating levels of PMVs may have a direct pathophysiological role in the development of several diseases and thus quantification/characterisation of PMVs may serve as an early diagnostic screening tool for those diseases [8]. PMVs are implicated in cardiovascular diseases as their composition and microRNA content are specific signatures of cellular activation and injury [28]. Also, the potential of PMVs in transferring biological information to neighbouring cells have given them an active role in inflammatory diseases, including atherosclerosis and angiogenesis. For example, they promote joint inflammation in rheumatoid arthritis by transporting proinflammatory cytokine interleukin-1 [29]. PMVs have again, been implicated in the process of remarkable anti-tumor reversal effect in cancer, tumor immune suppression, metastasis, tumor-stroma interactions and angiogenesis along with having a primary role in tissue regeneration [29]. Their levels have also been seen to be raised in thalassaemia and sickle cell diseases [11, 30].

Although increased PMVs, compared to healthy controls, have been associated with increased severity of diseases such as β-thallasaemia and Sickle Cell [11, 19], there is only evidence to support a circumstantial association at this stage as many studies are only quantitative and does not look much into the specific role of PMVs in disease states and their contribution to pathogenesis of the diseases [11, 30]. Hopefully, more experiments that focus on the function of PMVs in disease states will emerge in the future to give concrete evidence of the role PMVs play in diseases. A recent study on erythrocyte PMVs and other circulating PMV subtypes in sickle cell disease found that they did not detect Tissue Factor^+^ (TF^+^) PMVs, monocyte (CD14^+^) PMVs (MPMVs) or endothelial cell (CD 144^+^, CD146^+^, CD62E^+^) PMVs (EPMVs) in their samples [31]. Contrastingly, another group did not just detect TF^+^ PMVs, EPMVs and MPMVs, but concluded that the observed increase gave weight to the hypothesis that sickle cell disease is an inflammatory state with endothelial cell and monocyte activation along with abnormal vessel wall activity [32]. Unsurprisingly, both groups used different methods for PMV isolation [12], but as seen in Table 1, PMVs generally carry proteins (surface markers) characteristic of their parent cell.

**Table 1:** A table showing the cell of origin of PMVs and their characteristic surface markers

Cell of origin	Surface markers
Erythrocyte	CD 235a
Lymphocyte	CD3, CD4, CD8
Neutrophil/granulocyte	CD66b, CD66e
Monocyte	CD14
Platelet	CD41, CD42, CD61
Endothelial cell	CD105, CD144, CD62e

### PMVs and their constituents

Owing to the plasticity of the lateral organization of the plasma membrane into raft domains, known to segregate particular proteins and lipid species, a given stimulus can be expected to elicit the release of PMVs [33]. These PMVs may carry microRNA, mRNA, numerous membrane proteins, bioactive lipids and cytoplasmic constituents, characteristic of their parental cells which are implicated in a variety of fundamental processes [8]. PMVs also carry the majority of non-conventionally secreted proteins released into culture supernatants, and potentially carry them within the PMVs themselves [33]. This help in the export of proteins lacking a signal peptide as an alternative to conventional protein export. Amongst these, epimorphin, fibroblast growth factor 1 and 2 (FGF-1 and FGF-2), macrophage migration inhibitory factor (MIF) and galectin 3 (Gal-3) are all transported to the plasma membrane via the adenosine triphosphate cassette transport channel (ABCA1) needed for the release of PMVs or by exocytosis of exosomes [34].

The potential of PMVs to transmit proteins between cells play an important part in intercellular communication and the maintenance of homeostasis under physiological conditions, or initiation of deleterious processes in the case of excess or when carrying pathogenic constituents [6]. Most recently PMVs derived from embryonic stem cells (ESC) were found to carry Wnt-3, which is involved in haematopoietic differentiation and such PMVs were shown to reprogramme haematopoietic progenitor cells [6]. The ability of cells stimulated by sublytic complement to release PMVs has been demonstrated by work showing that these PMVs carry surface molecules such as TGF-β1 with which they in turn cause promonocytic/monocytic cells to differentiate along the monocyte/macrophage differentiation lineage (Table 2) [35]. PMVs can therefore be considered a disseminated storage pool of bioactive effectors, the nature and proportion of the latter accounting for duality, more particularly evidenced in vascular disease, inflammation, and immunity [36].

**Table 2:** A table with selected surface protein markers that enrich PMVs from circulation and the diseases they code for

Surface markers	Disease
L1CAM, CD24, ADAM10, EMMPRIN	Ovarian cancer
TGFβ1, MAGE3/6	Ovarian cancer
Claudin-4	Ovarian cancer
EGFRvIII	Glioblastoma
EGFR, EGFRvIII, PDPN, IDH1	Glioblastoma
CD63 and caveolin 1	Melanoma
FasL	Oral cancer
HER-2/neu, CCR6	Gastric cancer
Tissue factor (CD142)	Cancer-associated thrombosis
Amyloid B	Alzheimer disease
Wnt - 3	Leukaemia

## Phenotypical differences in PMVs from normal vs. cancer patients

PMVs in cancer patients were first documented in 1978, when they were identified in cultures of spleen nodules and lymph nodes of a male patient with Hodgkin disease [37]. The amount of PMVs shed by tumor cells has been shown to correlate with their invasiveness both *in vitro* and *in vivo* and there are phenotypical differences in PMVs isolated from normal as against cancer patients [38, 39]. Isolated PMVs from cancer patients tend to have a distinct molecular profile from that isolated from normal controls: in addition to conventional PMV markers (LAMP1 or MHC-I), they contain membrane-associated transforming growth factor-β1, FasL, MICA/MICB and myeloid blasts markers CD34, CD33 and CD117. These PMVs have the ability to decrease natural killer cell cytotoxicity and down-regulate the expression of NKG2-D in normal natural killer cells. In a recent study by Miroslaw J. Szczepanski et al, the PMVs fractions obtained from sera of AML patients had significantly greater protein content (75 ± 12 μg protein/mL) than those isolated from sera of normal controls (1.2 ± 0.4 mg protein/mL) [40]. Generally, PMVs derived from human cancer cells have received a good deal of attention because of their ability to participate in the horizontal transfer of signalling proteins between cancer cells and contribute to their invasive activity.

### Therapeutic significance of PMVs

Taking into consideration the ability of PMVs to transmit information between cells through proteins, lipids and nucleic acids (DNA, mRNA, microRNA) transfer and on their ability to create a communication network between cells, it is plausible that they could serve as tools with veritable therapeutic potential [41]. In this context, ligands carried by PMVs can directly interact with receptors in target cells and induce signal transduction. In addition, membranes of PMVs can fuse with the plasma membrane of target cells, leading to the transfer of membrane components and delivery of PMV cytoplasmic content [42]. This can result in the activation or inhibition of intracellular pathways of target cells or in the modification of their phenotype [43]. Through their action, PMVs have a great effect on angiogenesis. This is because they can induce the production and transfer of proangiogenic or antiangiogenic factors, modify endothelial cell function concerning adhesion, migration, or proliferation - the 3 key steps in the formation of new vessels (angiogenesis) and subsequently promote cancer progression as well as the pathogenesis of other diseases [44, 45]. Cancer progression is dependent on abnormal angiogenesis, in particular, when exacerbated neovascularization forms new blood vessels that supply adequate nutrients, oxygen, and growth factors to facilitate the growth of the tumor and metastasis development [46]. Tumor angiogenesis therefore results from an imbalance of proangiogenic and antiangiogenic factors released from tumor cells and circulated by PMVs [47].

Tumour cells can produce PMVs constitutively without any apparent need for stimulus but vesiculation can be increased by stress, including exposure to chemotherapeutic drugs and heat [48]. Cancer patients not only exhibit tumour -derived PMVs but also high levels of platelet-derived PMVs and the hypercoagulation typically observed with malignancy can be attributed in part to these procoagulant PMVs [49]. It has also been postulated that tumour cells can evade apoptosis by releasing PMVs that contain caspase-3, thereby preventing its intracellular accumulation [50]. Inhibition of this release has been shown to result in caspase-3 accumulation and subsequent apoptosis [50]. Again, metastatic cancer cells can degrade the extracellular matrix (ECM) through the transfer of surface proteases such as matrix metalloproteinases (MMPs), urokinase-type plasminogen activator (uPA) and cathepsins via PMVs [51]. Tumour cells have been shown to release PMVs rich in MMPs and uPA, which degrade the ECM, allowing for cell invasion [52].

There is also the PMVs assisted dissemination of cancer multidrug resistance, by which drug sensitive cells can acquire the resistance phenotype via long-range communication [53, 54]. Multi-drug resistance can also come from the expulsion of therapeutic drugs from tumor cells through PMVs release [54]. In a demonstration, tumor cells treated with doxyrubicin accumulated and released the drug in shed PMVs, implying PMV shedding as a drug-efflux mechanism involved in drug resistance [55]. By virtue of their ability to harness select bioactive molecules and propagate the horizontal transfer of these cargoes, shed PMVs can have an enormous impact on tumor growth, survival and spread [55]. PMVs have also been implicated in the pathogenesis of thrombosis, diabetes, inflammation, atherosclerosis and vascular cell proliferation. From evidences, as outlined above, it implies that PMVs are important mediators of cell communication and vital components of the tumor microenvironment niche as well as in the pathogenesis of other diseases.

## Conclusion

Since knowing the characteristics of PMVs are so important, there have been various studies undertaken in this regard in trying to come out with a set of standards by which they can be measured, such as the appropriate centrifuge speed, sample removal and storage, flow cytometry (types and mode of operation) amongst others to offset the various pre-analytical variables known. It is also important to note that flow technology and its incorrect implementation could lead to result variability as well and therefore requires standardisation. This review discussed typical characteristics of PMVs and their therapeutic significance which could be a bench mark for PMVs analysis as we move to the era of using PMVs in diagnosing diseases and as a therapeutic tool. It is important that further studies are carried out to ascertain the functions of the characteristics discussed which will lead to understanding the general role of PMVs both in health and disease states.

## Abbreviations

ABCA1: ATP-binding cassette transporter A1
AML: Acute myelocytic leukaemia
CD: Cluster of differentiation
DNA: Deoxyribonucleic acid
ECM: Extracellular matrix
ELISA: Enzyme linked immunosorbent assay
ESC: Embryonic stem cell
FACS: Fluorescence activated cell sorting
FasL: Fas ligand
FGF: fibroblast growth factor
Gal-3: Galectin - 3
LAMP-1: Lysosomal-associated membrane protein 1
MHC: Major histocompatibility complex
MICA: MHC class I polypeptide-related sequence A
MICB: MHC class I polypeptide-related sequence B
MIF: Macrophage migration inhibitory factor
MMPs: Matrix metalloproteinases
MPs: Microparticles
MVB: Multivesicular bodies
MVs: Microvesicles
NKG2-D: Natural Killer group 2 - D
PMVs: Plasma Membrane-derived Vesicles
RNA: Ribonucleic acid
TF: Tissue Factor
TGF – β1: Tissue Growth Factor – beta 1
uPA: Urokinase-type plasminogen activator
